# Deficiency of Huntingtin Has Pleiotropic Effects in the Social Amoeba *Dictyostelium discoideum*

**DOI:** 10.1371/journal.pgen.1002052

**Published:** 2011-04-28

**Authors:** Michael A. Myre, Amanda L. Lumsden, Morgan N. Thompson, Wilma Wasco, Marcy E. MacDonald, James F. Gusella

**Affiliations:** 1 Molecular Neurogenetics Unit, Center for Human Genetic Research, Massachusetts General Hospital, Boston, Massachusetts, United States of America; 2 Genetics and Aging Research Unit, MassGeneral Institute for Neurodegenerative Disease, Massachusetts General Hospital, Charlestown, Massachusetts, United States of America; Stanford University School of Medicine, United States of America

## Abstract

Huntingtin is a large HEAT repeat protein first identified in humans, where a polyglutamine tract expansion near the amino terminus causes a gain-of-function mechanism that leads to selective neuronal loss in Huntington's disease (HD). Genetic evidence in humans and knock-in mouse models suggests that this gain-of-function involves an increase or deregulation of some aspect of huntingtin's normal function(s), which remains poorly understood. As huntingtin shows evolutionary conservation, a powerful approach to discovering its normal biochemical role(s) is to study the effects caused by its deficiency in a model organism with a short life-cycle that comprises both cellular and multicellular developmental stages. To facilitate studies aimed at detailed knowledge of huntingtin's normal function(s), we generated a null mutant of *hd*, the *HD* ortholog in *Dictyostelium discoideum*. *Dictyostelium* cells lacking endogenous huntingtin were viable but during development did not exhibit the typical polarized morphology of *Dictyostelium* cells, streamed poorly to form aggregates by accretion rather than chemotaxis, showed disorganized F-actin staining, exhibited extreme sensitivity to hypoosmotic stress, and failed to form EDTA-resistant cell–cell contacts. Surprisingly, chemotactic streaming could be rescued in the presence of the bivalent cations Ca^2+^ or Mg^2+^ but not pulses of cAMP. Although *hd* ^−^ cells completed development, it was delayed and proceeded asynchronously, producing small fruiting bodies with round, defective spores that germinated spontaneously within a glassy sorus. When developed as chimeras with wild-type cells, *hd* ^−^ cells failed to populate the pre-spore region of the slug. In *Dictyostelium*, huntingtin deficiency is compatible with survival of the organism but renders cells sensitive to low osmolarity, which produces pleiotropic cell autonomous defects that affect cAMP signaling and as a consequence development. Thus, *Dictyostelium* provides a novel haploid organism model for genetic, cell biological, and biochemical studies to delineate the functions of the HD protein.

## Author Summary

Genetic evidence in humans and mouse models of Huntington's disease suggests that the disease mutation confers a deleterious gain-of-function on huntingtin that acts through the deregulation of some aspect of the protein's normal function(s). While huntingtin's function is poorly understood, its evolutionary conservation makes investigation of its physiological role in lower organisms an attractive route that has yet to be fully exploited. Therefore, we have used *Dictyostelium discoideum* to study the consequences of huntingtin (*hd*) deficiency. Developing *Dictyostelium* cells chemotax to form a multicellular slug that forms a fruiting body, comprising dormant spores encased above dead stalk cells. We found that *hd* ^−^ cells were hypersensitive to hypoosmotic stress. When starved, *hd* ^−^ cells aggregate by accretion, showed disorganized F-actin, and failed to form EDTA-resistant cell–cell contacts. Surprisingly, chemotactic signaling was rescued with Ca^2+^ or Mg^2+^ but not pulses of cAMP. Development of *hd* ^−^ mutants produced small fruiting bodies with round, defective spores, and when mixed with wild-type cells they didn't differentiate into spores. Our results are consistent with mammalian studies that show huntingtin is a multifunctional protein involved in many biochemical processes; and, importantly, they establish *Dictyostelium* as a valuable experimental organism for exploring in biochemical detail huntingtin's normal function(s).

## Introduction

Huntington's disease (HD) is an autosomal dominant neurodegenerative disorder whose clinical manifestations include psychiatric disturbances, cognitive decline and characteristic involuntary movements, typically diagnosed in mid-life [1], [2]. HD is caused by a CAG trinucleotide repeat expansion mutation (>35 units) that produces an elongated version of a normally polymorphic polyglutamine segment in huntingtin [3]–[5], a large 350 kDa ubiquitously expressed HEAT (*h*untingtin, *e*longation factor 3, the *A* subunit of protein phosphatase 2A, and *T*OR1) repeat protein.

Several lines of evidence indicate that the HD mutation confers a CAG length-dependent ‘gain-of-function’ that likely produces its distinctive neuropathology through a modulatory effect on some structural or functional feature of huntingtin [6]–[9]. HD homozygote individuals with two mutant *HD* genes, and therefore no wild-type huntingtin, develop the characteristic movement disorder with timing comparable to that seen in typical *HD* mutation heterozygote individuals, indicating the absence of a strong dosage effect. Moreover, complete deficiency of huntingtin causes developmental abnormalities and embryonic lethality in the mouse that can be fully rescued by mutant huntingtin, indicating that these fundamental normal functions of huntingtin are not abrogated by the HD mutation. Thus, defining the disease-producing ‘gain-of-function’ - either a polyglutamine-length dependent increase or deregulation of a normal huntingtin activity or the introduction of a novel polyglutamine-length dependent activity, will require an understanding of the protein's normal function(s).

Huntingtin is present throughout eukaryotic evolution except in fungi and plants and shows no close primary sequence homology to any other protein [6]. Therefore, one approach to huntingtin function is to investigate its orthologs in tractable experimental models. Manipulation of the *HD* gene homologs in model organisms has revealed that huntingtin is essential for normal embryonic development both in the mouse and in the zebrafish [10]–[13], is dispensable for *Drosophila* development [14] and is implicated in a variety of functions ranging from vesicle trafficking to chromatin silencing and gene expression [15]–[18]. However, though murine embryonic stem cells lacking huntingtin are viable in tissue culture, permitting multi-cellular developmental studies *in vitro* [10], investigation of huntingtin function in single cells versus multicellular stages of development would be expedited by an experimental organism with a short life-cycle, comprising cellular and multicellular developmental stages.

The soil amoeba *Dictyostelium discoideum* is a *bona fide* multicellular eukaryotic organism with a haploid genome and a relatively simple developmental program that serves as a model for basic biological research [19] and is emerging as a valuable tool for understanding gene function and pathogenic mechanisms in a variety of human disorders [20]–[24]. During development, *Dictyostelium* undergoes a series of coordinated morphological and physiological changes that are initiated by starvation and progress in defined stages over a 24 hour period. Within the first 6 hours of development, cells secrete, and undergo chemotaxis toward cyclic adenosine monophosphate (cAMP) to form aggregation centers. The secretion of cAMP promotes a G protein-coupled receptor signal/response cascade resulting in the formation of loose mounds comprising up to 100,000 cells [25], [26]. As development continues, cells within the mound are directed to differentiate into either prestalk or prespore cells, leading to morphological changes that yield a multicellular stalk, supporting a ball of encapsulated dormant spores [27].

To explore huntingtin function in both single cells and multicellular structures of the same organism, we have characterized a *Dictyostelium* ortholog (*hd*) of the human *HD* gene, have generated a viable *hd*-null mutant and have delineated a number of consequent phenotypes. We present evidence to suggest that huntingtin is a multifunctional protein that plays a protective role during hypoosmotic stress, regulates cell shape and homophilic cell-cell adhesion through a cytoskeletal–based mechanism, modulates pre-spore/spore cell fate determination and affects spore dormancy in *Dictyostelium*. Interestingly, *hd* ^−^ cells were found to have a requirement for bivalent cations in the medium for optimal chemotaxis and cell aggregation. Our findings establish *Dictyostelium* as a valuable experimental eukaryotic organism for exploring in biochemical detail huntingtin's normal function(s), as defined by the pleiotropic effects of huntingtin deficiency throughout the developmental life cycle.

## Results

### The *Dictyostelium* huntingtin ortholog

**Figure 1:**
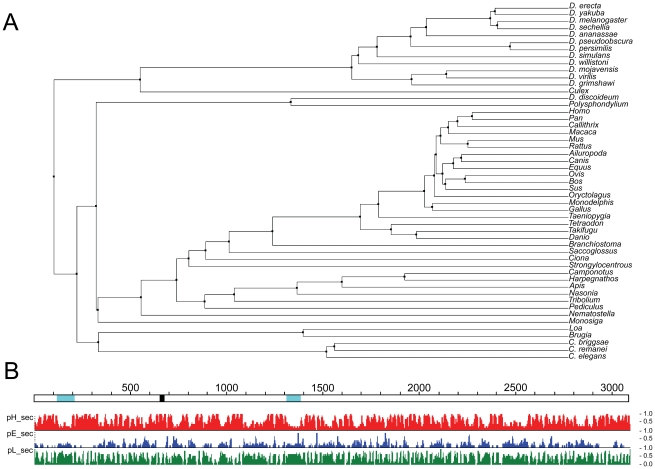
Phylogenetic and structural analysis of *Dictyostelium* huntingtin. A. A phylogenetic tree showing the relationship of *Dictyostelium* huntingtin to huntingtin proteins from 51 other organisms was prepared as the average distance tree using BLOSUM62 of a CLUSTALW alignment in JALVIEW (reference: Waterhouse, A.M., Procter, J.B., Martin, D.M.A, Clamp, M. and Barton, G. J. (2009) “Jalview Version 2 - a multiple sequence alignment editor and analysis workbench” Bioinformatics 25 (9) 1189–1191). B. *Dictyostelium* huntingtin (open line) is depicted with amino acid coordinates (above) above secondary structure predictions from PROF (www.predictprotein.org; B Rost, G Yachdav and J Liu (2004) The PredictProtein Server. Nucleic Acids Research 32(Web Server issue):W321–W326) where pHsec, pEsec and pLsec represent the probability (1 high, 0 low) for helix (red), strand (blue) and neither helix nor strand (green). Locations in the huntingtin schematic of natively unstructured regions predicted by NORSnet (Avner Schlessinger and Jinfeng Liu and Burkhard Rost (2007)) and of a 19 residue polyglutamine repeat are shown by cyan and black, respectively.

The *Dictyostelium discoideum* genome, examined via dictyBase (www.dictybase.org) [28], contains a single gene (DDB_G0272344) with evident sequence homology to human huntingtin. The *hd* locus is comprised of four exons, located on chromosome 2. Analysis of GenBank with psi-BLAST [29], [30] placed the product of *hd* firmly within the huntingtin family (Figure 1A), with a length of 3,095 amino acids comparable to the 3,144 amino acids of human huntingtin. Structural analysis of huntingtin proteins has identified the presence of numerous HEAT and HEAT-like repeats thought to produce a large α-helical solenoid rather than a typical globular protein [6], [15], [31], [32]. *Dictyostelium* huntingtin is also predicted to be α-helical across most of its length (Figure 1B) and, as in other organisms, BLASTP searches of GenBank revealed no significant sequence alignments with other *Dictyostelium* proteins. Interestingly, *Dictyostelium* huntingtin contains a polyglutamine tract of 19 residues (Figure 1B) that is comparable in size to that found in normal-range human huntingtin but is encoded by the trinucleotide repeat CAA interrupted by a single CAG codon, is not followed by a polyproline domain, is located further downstream of the initiator methionine (at residue 533 rather than residue 18 as in the human protein), and may reflect the unusually high number of predicted proteins (∼34%) that contain homopolymer tracts of 15 residues or more in *Dictyostelium* [33].

### *Dictyostelium* huntingtin was expressed throughout growth and development

**Figure 2:**
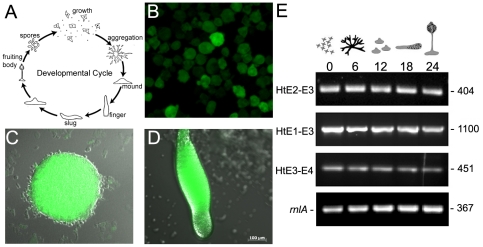
The predicted endogenous hd promoter drives expression of GFP during growth and development. (A) Developmental life cycle of Dictyostelium discoideum*. *Dictyostelium* spends most of its life as vegetative cells that prey upon bacteria and divide mitotically. When starved, the cells enter the developmental cycle that completes in a 24 hour period. During development, amoebae chemotactically aggregate towards secreted cAMP to form a hemispherical mound. Within the mound cells differentiate and sort out to form a motile slug. The slug ultimately forms a fruiting body comprised of a multicellular stalk supporting a ball of encapsulated dormant spores. (B) A cloned region of DNA sequence 595 bp upstream of the *hd* gene was used to replace the actin 15 promoter in the expression vector pTX-GFP. Wild-type AX3 cells were transformed with this construct and expression of GFP was assessed in vegetative cells; (C) mound structure showing GFP expression from *hd* promoter; (D) slug showing expression of GFP in both prestalk (anterior) and prespore (posterior) regions. Scale bar (µm) is shown at the *bottom right*. (E) *Dictyostelium* huntingtin is a single copy gene comprised of four exons. Total RNA was extracted from wild-type *Dictyostelium* strain AX3 during vegetative growth and 6-hour increments during synchronous development on nitrocellulose filters supported by filter pads soaked in DB buffer and developed at 21°C. The presence of alternate transcripts was analyzed with a combination of *hd* exon-specific primer sets, RT-PCR and resolved using ethidium bromide stained agarose gels. *rnlA* was used as a mRNA amplification control. Collection time points are shown in hours (*top*). *hd* exon boundaries are shown *on the left*. PCR product sizes (bp) are shown *on the right*. *Figure representing the developmental life cycle of *Dictyostelium* was adapted from *CC Creative Commons Attribution – Share Alike 3.0, David Brown & Joan E. Strassmann* and is freely available online for download at the dictyBase.

To determine the spatial and temporal pattern of huntingtin mRNA expression throughout the life cycle (Figure 2A); we cloned the intergenic region up to the closest upstream gene (608 bp) and used it to replace the actin15 promoter in pTX-GFP and to direct expression of the green fluorescent protein (GFP), which was detected in all cell types of transformed AX3 wild-type *Dictyostelium* cells during growth and development (Figure 2B, 2C, 2D), a pattern that fits well with the developmental and cell-type specific mRNA expression data acquired through extensive microarray and RNA-seq analysis which is freely available in dictyBase (DDB_G0272344) [34]. To further characterize the function of huntingtin in *Dictyostelium*, we examined the existence of alternate transcripts using exon specific primers and reverse transcriptase polymerase chain reaction (RT-PCR). No alternatively spliced forms of mRNA could be detected using various primer sets specific for exons (exon 1–exon 4) during vegetative growth and development of AX3 cells (Figure 2E).

### Huntingtin-null cells display pleiotropic phenotypes

**Figure 3:**
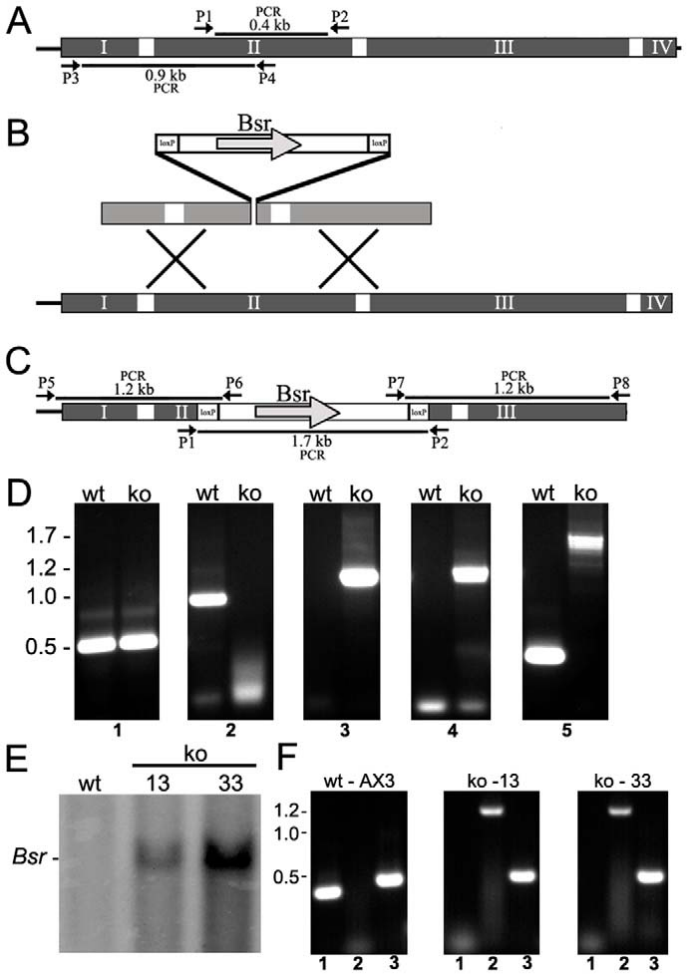
Targeted disruption of the *hd* gene by homologous recombination. (A) Physical map of the *hd* gene is shown. The four exons (*I, II, III and IV*) that comprise the *hd* gene are shown (*grey*) interrupted by three introns (*white* bars). PCR primers are shown as *arrows* at the positions in which they prime including amplicon sizes. The exon-intron boundaries are not to drawn to scale. (B) The targeting vector and sites of recombination are shown. The vector contains the Blasticidin S resistance gene (*Bsr*) that is flanked by loxP sites and allows for selection of transformed cells. (C) Physical map of the targeted deletion is shown. PCR primers are shown as *arrows* at the positions that they prime including amplicon sizes. (D) Genomic PCR of wild-type (*wt*) and *hd* ^−^ (*ko*) clones. Control PCR amplification of the sequence immediately upstream of the *hd* gene (*panel 1*); No amplification in clones carrying a targeted deletion of the *hd* gene which removes 278 bp of sequence from exon 2 (*panel 2*); PCR amplification of genomic DNA using primers that prime outside the vector (P5 or P8) and inside the *Bsr* cassette (P6 or P7), respectively (*panels 3 and 4*). No DNA is amplified from wild-type cells; PCR of genomic sequences that flank the insertion site (*panel 5*). The endogenous wild-type allele yields a fragment of ∼400 bp whereas the knockout mutant yields a much larger fragment representing insertion of the *Bsr* cassette. Molecular weight markers are shown *on the left*. (E) Confirmation of gene disruption by Southern blot analysis. Genomic DNA from wild-type or two independent *hd* ^−^ clones (clones 13 and 33) were probed by Southern blot hybridization for the presence of *Bsr* sequences. (F) RT-PCR of total RNA isolated from wild-type and two independent *hd* ^−^ clones (clones 13 and 33). In each panel, *lane 1* represents the amplicon derived from priming the exon 2 deletion; lane 2 represents priming inside the *Bsr* cassette; and *lane 3* represents control RT-PCR reactions. Molecular weight markers are shown *on the left*.

To explore huntingtin function, we utilized homologous recombination to create multiple independent *Dictyostelium* strains carrying a disruption of the *hd* gene in the haploid genome (Figure 3A). The disruption cassette targeted *hd* such that a double-recombination event would remove 278 bp of exon 2 and insert a blasticidin resistance (*Bsr*) selection cassette (Figure 3B, 3C). Following transformation of parental AX3 cells and blasticidin selection, 150 independent resistant clones were isolated and those (16) with the properly targeted *hd* gene disruption were identified by PCR amplification of the expected genomic DNA products (Figure 3D). Proper targeting was also confirmed by Southern blot using DIG-labeled *Bsr* specific probes as well as by RT-PCR amplification of cellular mRNA which demonstrated that *hd* mRNA was absent (Figure 3E, 3F). Clonal isolates from multiple independent mutants possessing the same disruption and developmental phenotype (glassy sori and round spores) were obtained. One of these clones (httE13) was used in all subsequent experiments and is referred to as *hd* ^−^ for clarity.

### Actin cytoskeleton and contractile vacuole defects in *hd* ^−^ cells

**Figure 4:**
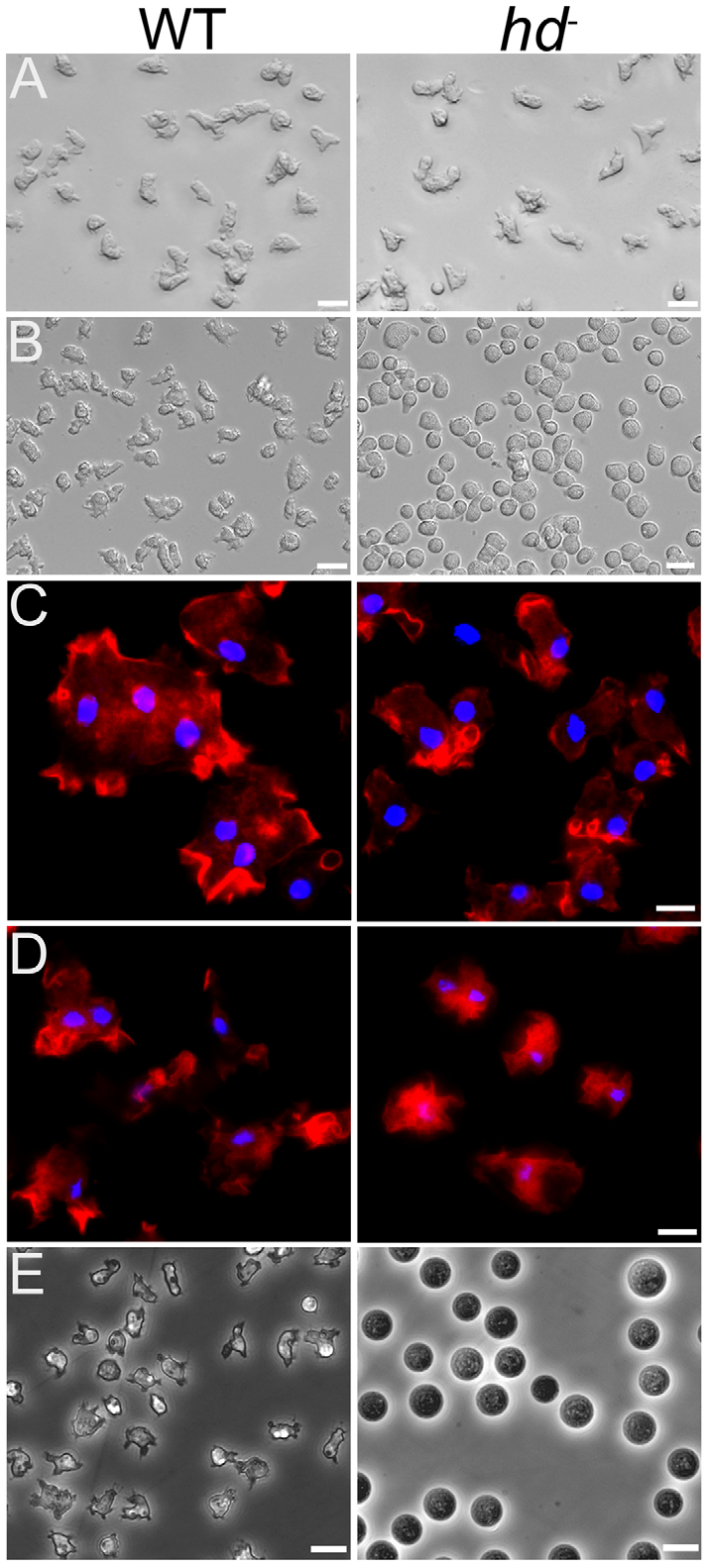
Removal of nutrients caused rounding of *hd* ^−^ cells and affects F-actin localization. (A) The morphology of *hd* ^−^ cells (*right panel*) grown and photographed as an adherent culture in HL-5 was similar to wild-type cells (*left panel*). Scale bars (20 µm) are shown on the *bottom right*. (B) Removal of nutrients (HL-5) and replacement with starvation buffer (KK_2_) caused rounding of *hd* ^−^ cells. Cells were photographed 20 minutes after the addition of starvation buffer. Wild-type cells (*left panel*) displayed morphology similar to that observed for cells in HL-5 whereas all *hd* ^−^ cells immediately adopted a rounded morphology (*right panel*). Scale bars (20 µm) are shown on the *bottom right*. (C) Aberrant localization of F-actin in starved *hd* ^−^ cells. Vegetative wild-type cells (*left panel*) and *hd* ^−^ cells (*right panel*) collected from axenic medium (HL-5) were deposited into Lab-tek chambered cover glass (8 well) at 1×10^5^ cells/cm^2^ and allowed to grow overnight in HL-5 at 21°C. For F-actin staining, the cells were fixed with 4% formaldehyde in PDF buffer and then stained with Texas red-phalloidin. Arrows indicate membrane extensions enriched with F-actin. (D) Localization of F-actin in cells submerged in starvation buffer (KK_2_). One hour after starvation in KK_2_, wild-type cells retained their normal amoeboid morphology (*left panel*); *hd* ^−^ cells remained rounded and exhibited a redistribution of F-actin away from the cell cortex as it concentrated in the cytosol (*right panel*). Nuclei were stained with Hoechst 33342. Scale bars (5 µm) are shown on the *bottom right*. (E) *hd* ^−^ mutants were impaired in osmoregulation. Wild-type (wt) and (F) huntingtin-null (*hd* ^−^) cells were grown in cell culture dishes with HL-5 media. The media was exchanged with dH_2_O and the cells were examined after 1 hour. Wild-type cells initially rounded and showed partial swelling under the hypoosmotic conditions, but quickly recovered the ability to crawl and change shape. In contrast, *hd* ^−^ cells were completely round and swollen in dH_2_O, and barely remained attached to the culture dish. The *hd* ^−^ cells began to rapidly lyse after ∼3 hours of this treatment and underwent complete lysis by 6 hours suggesting that the mutants may retain some very low level of osmoregulation. Scale bar 10 µm.

We did not observe differences in the growth properties of *hd* ^−^ and wild-type cells when grown as adherent cultures in tissue culture dishes (data not shown). However, in contrast to wild-type cells, axenically grown shaking cultures of *hd* ^−^ cells consistently grew faster with a doubling time of ∼10 hours compared to the ∼12 hour doubling time of wild-type cells (Figure S1), suggesting a physiological impact of the absence of huntingtin at the level of single cells. Plating of *hd^−^* cells under non-nutrient, low ionic strength phosphate buffer (KK_2_; ∼40 mOsmol/L), elicited a cell rounding phenotype that suggested an actin-cytoskeleton defect, which we examined by staining for F-actin. The morphology of *hd* ^−^ cells grown as an adherent culture in HL-5 medium was similar to that of wild-type cells (Figure 4A) and phalloidin staining showed a similar distribution of F-actin in extended pseudopods in both genotypes (Figure 4C). However, removal of nutrients and the addition of KK_2_ buffer resulted in a rapid reduction in pseudopod formation (∼20 minutes) and ultimately a failure to polarize only in the *hd* ^−^ cells (Figure 4B). The rounded cell phenotype of *hd* ^−^ cells was accompanied by a redistribution of F-actin from the cell cortex to the cytosol (Figure 4D).

As the extreme cell rounding observed in low osmotic buffer hinted at a potential defect in contractile vacuole osmoregulation, we assessed the ability of *hd* ^−^ cells to respond to osmotic stress. When wild-type cells were placed in water, several small contractile vacuoles formed within 30 minutes and, over time, the cells compensated for the sudden change in their osmotic environment and gradually regained their original size, shape and cell-substratum adhesion (Figure 4E; Video S1). In contrast, *hd* ^−^ cells failed to form any visible vacuoles, but instead became round, swollen, lost their adhesion to the substratum and underwent complete lysis within 5–6 hours (Figure 4F; Video S2), indicating that contractile vacuole activity was dramatically compromised in *hd* ^−^ cells under conditions of hypotonic stress. No difference was observed between wild-type and *hd^−^* cells under hyperosmotic (400 mM sorbitol) conditions (data not shown).

### Loss of huntingtin caused developmental delay and abnormal morphogenesis

**Figure 5:**
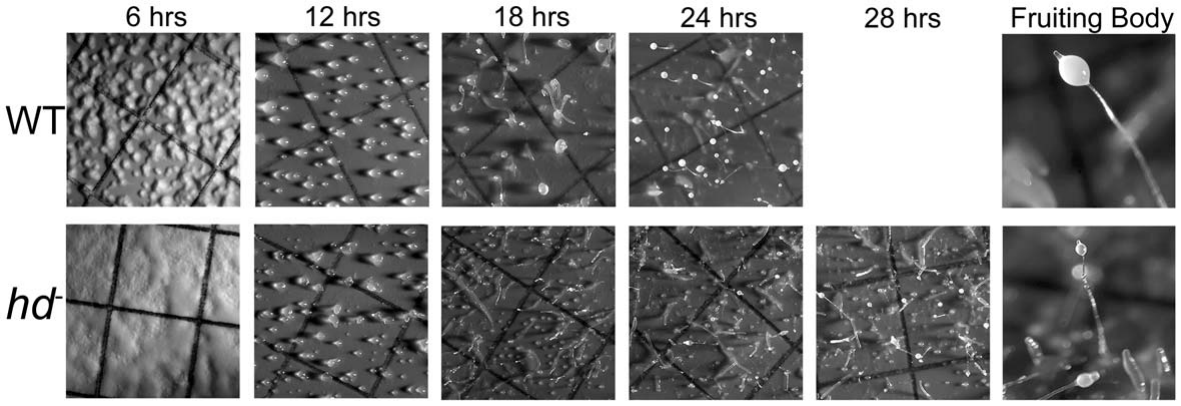
*hd* ^−^ mutants exhibited asynchronous delayed development. Exponentially growing wild-type and *hd* ^−^ cells (2×10^6^ cells/mL) were washed three times with DB buffer, resuspended at a density of 5×10^7^ cells/mL, deposited on black nitrocellulose filters supported by filter pads soaked in DB buffer and developed for 28 hours at 21°C. All photographs are top view. Wild-type cells developed distinct aggregation territories after 6 hours whereas *hd* ^−^ null cells displayed a much smoother appearance and the noticeable absence of streams. By 12 hours *hd* ^−^ cells formed mounds that tended to produce multiple tips. Further development of *hd* ^−^ cells proceeded in a delayed asynchronous manner resulting in the presence of various intermediate structures and small aborted mounds. The developmental time points in hours are shown *at the top*.

To determine whether the mutant cells were capable of carrying out the full program of multicellular development, we assayed wild-type and *hd* ^−^ cells by plating on filters. Wild-type cells formed numerous streaming aggregation centers by 6 hours that went on to form tight mounds by 12 hours, early culminants by 18 hours and fruiting bodies by 24 hours as expected (Figure 5). By contrast, *hd* ^−^ mutant cells formed delayed aggregates, and by 12 hours had constructed comparatively loose mounds with supernumerary prestalk tips, which subsequently went on to form multiple slugs and small fruiting bodies (Figure 5). Thus, whereas wild-type cells had completed the developmental program by ∼24 hours, the development of *hd* ^−^ cells was delayed and asynchronous, resulting in the appearance of aborted intermediates and the formation of small fruiting bodies that develop glassy sori.

### Huntingtin-deficient cells exhibited abnormal shape and motility during early development

**Figure 6:**
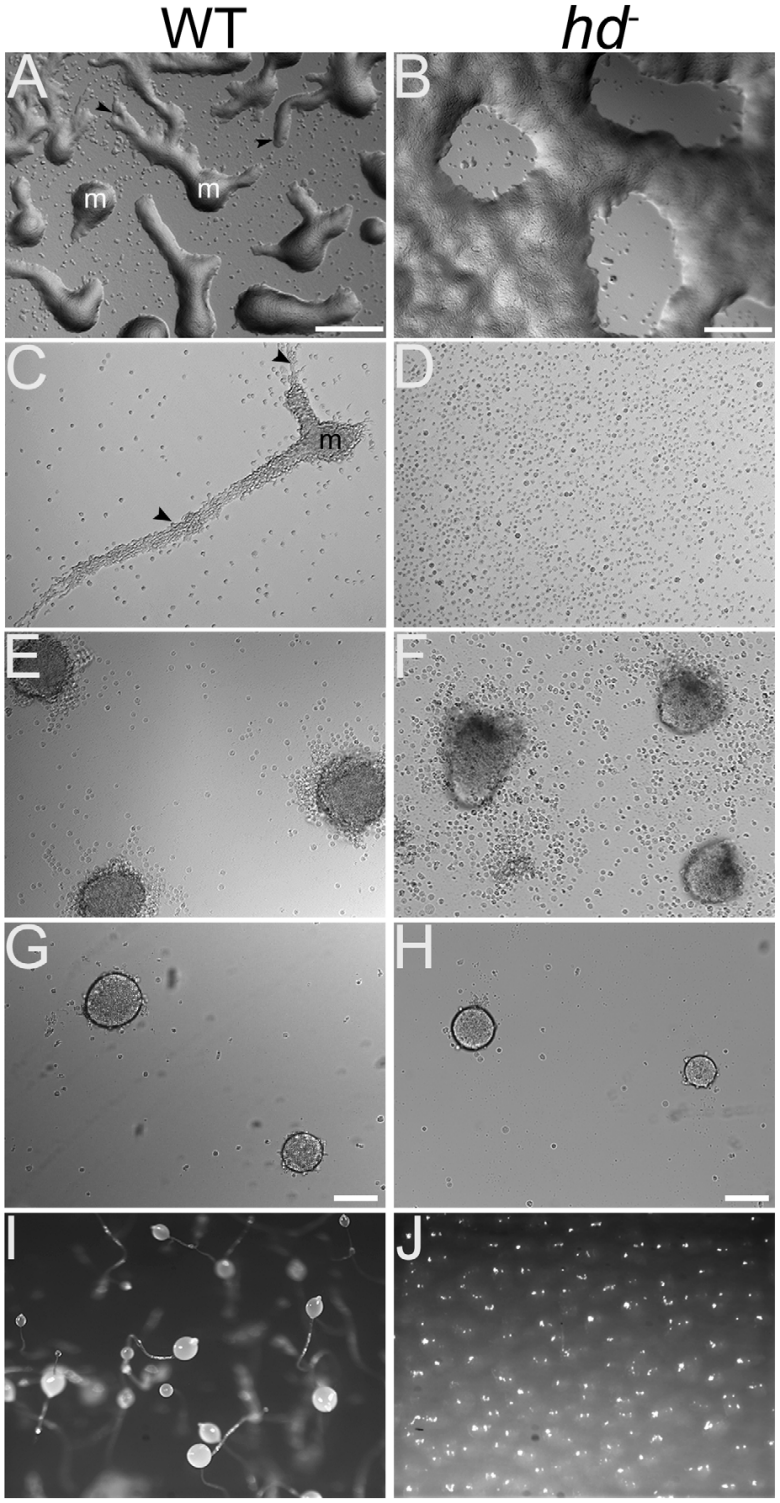
*hd* ^−^ cells displayed aggregation defects and failed to stream under submerged culture. Wild-type and *hd* ^−^ cells (1×10^6^ cells/mL) were deposited on non-nutrient KK_2_ agar plates and visualized by brightfield microscopy. Images are top view. (A) Wild-type cells migrated in streams (*arrows*) to form aggregation territories (*m*) by ∼6 hours. (B) *hd* ^−^ cells did not form well-defined streams, but rather clusters of cells that formed aggregation centers largely by accretion. Scale bar 100 µm. (C) Wild-type and (D) *hd* ^−^ cells (1×10^5^ cells/cm^2^) were submerged under KK_2_ and allowed to develop for 6 hours. Streams (*arrows*) of wild-type cells moving into an aggregation center (*m*) were readily apparent (*left panel*) whereas *hd* ^−^ cells failed to stream (*right panel*). (E) Wild-type and (F) *hd* ^−^ cells were submerged under buffer at high density (5×10^6^ cells/cm^2^) and imaged after 20 hours. Under these conditions, wild-type cells readily formed distinct organized mounds; high density partially restored the ability of *hd* ^−^ cells to form aggregation territories but with an irregular polarized shape. (G) Wild-type and (H) *hd* ^−^ cells (1×10^5^ cells/cm^2^) were submerged under KK_2_ in the presence of 1 mM CaCl_2_ and imaged after 20 hours. Under these conditions exogenous calcium rescues early development of *hd* ^−^ cells. Scale bar 100 µm. (I) Development on KK_2_ agar in the presence of EGTA. After 24 hours, wild type cells form fruiting bodies whereas development of *hd* ^−^ cells (J) is blocked in the presence of 1 mM EGTA.

A closer examination of the effects that loss of huntingtin has on the earliest stages of development revealed deficits in both the aggregation properties and cell-cell cohesiveness of wild-type and *hd* ^−^ cells. When assayed on non-nutrient KK_2_ agar or buffer-soaked nitrocellulose filters, wild-type cells migrated as streams to form aggregates by 6–8 hours (Figure 6A). The *hd* ^−^ cells did not form streams that led to distinct aggregation territories, but rather form delayed aggregation territories by accretion (Figure 6B). We next examined the behavior of cells deposited at different cell densities under starvation buffer (non-nutrient phosphate buffer KK_2_, pH 6.2) in order to get a better view of the streaming defect. When plated at 1×10^5^ cells/cm^2^, wild-type cells formed long streams of polarized, elongated cells that congregated into aggregation signaling centers (Figure 6C; Video S3). In contrast, *hd* ^−^ cells adopted the rounded shape noted previously and ultimately failed to polarize, form aggregation centers or initiate streams (Figure 6D; Video S4). Yet, when assessed at high cell densities (5×10^6^ cells/cm^2^), *hd* ^−^ cells managed to form aggregation centers, largely by accretion; however the mounds appear polarized (regular shape on one side, irregular with dark clumping cells on the other side) and many cell were excluded (Figure 6E, 6F).

The observed defects suggest two possibilities; 1) *hd* ^−^ cells are defective in cAMP signaling, or 2) the extreme sensitivity of *hd* ^−^ cells to conditions of low osmolarity negatively affects development. The secretion of periodic cAMP pulses is required to initiate *Dictyostelium* development [35] and so the addition of timed pulses of exogenous cAMP should facilitate early development in *hd* ^−^ cells if they are defective in cAMP signaling. Pulsing wild type cells in KK_2_ buffer with 75 nM cAMP every 6 minutes for a period of 4–5 hours accelerated the onset of chemotactic streaming, but equivalent cAMP pulses did not rescue the rounding or chemotactic streaming of *hd* ^−^ cells (data not shown). We next assessed what effect the addition of bivalent cations (higher osmolarity) would have on cAMP signaling and chemotactic aggregation of *hd* ^−^ cells under KK_2_ buffer. Interestingly, the addition of 1 mM CaCl_2_ (Figure 6G, 6H), 1 mM MgCl_2_ or 1 mM MgSO_4_^−^ (data not shown) rescued endogenous cAMP signaling and chemotactic aggregation of *hd* ^−^ cells (Video S5). However, the streams formed by *hd* ^−^ cells in the presence of bivalent cations were shorter and had a tendency to break and re-form when compared to wild type streams (Figure S2A, S2B). In addition, rescue of cAMP signaling and chemotactic aggregation of *hd* ^−^ cells in the presence of bivalent cations was inhibited by the addition of 1 mM ethylene glycol tetraacetic acid (EGTA) (Video S6).

To further assess cAMP signaling in *hd* ^−^ cells, we explored their aggregation properties on KK_2_ agar containing caffeine (1, 2.5 and 5 mM), a compound shown to inhibit cAMP signaling in *Dictyostelium* [36]. *Hd* ^−^ mutant cells were equally sensitive and exhibited similar developmental phenotypes to wild type cells when treated with caffeine. Aggregation and development of wild type and *hd* ^−^ cells was severely inhibited at 5 mM caffeine and at 1 mM caffeine both wild type and *hd* ^−^ cells were capable of forming fruiting bodies (data not shown). We next assessed aggregation and development of wild type and *hd* ^−^ cells on KK_2_ agar containing EGTA (1 mM or 2 mM). In contrast to caffeine treatment, development of wild type cells in the presence of 1 mM EGTA was unaffected (Figure 6I) whereas *hd* ^−^ cells failed to form chemotactic streams and arrested development as loose aggregates (Figure 6J). Taken together, our data suggest that *hd* ^−^ cells are not primarily defective in cAMP signaling but rather have a hypersensitivity to conditions of low osmolarity which affects cAMP signaling and thus development.

**Figure 7:**
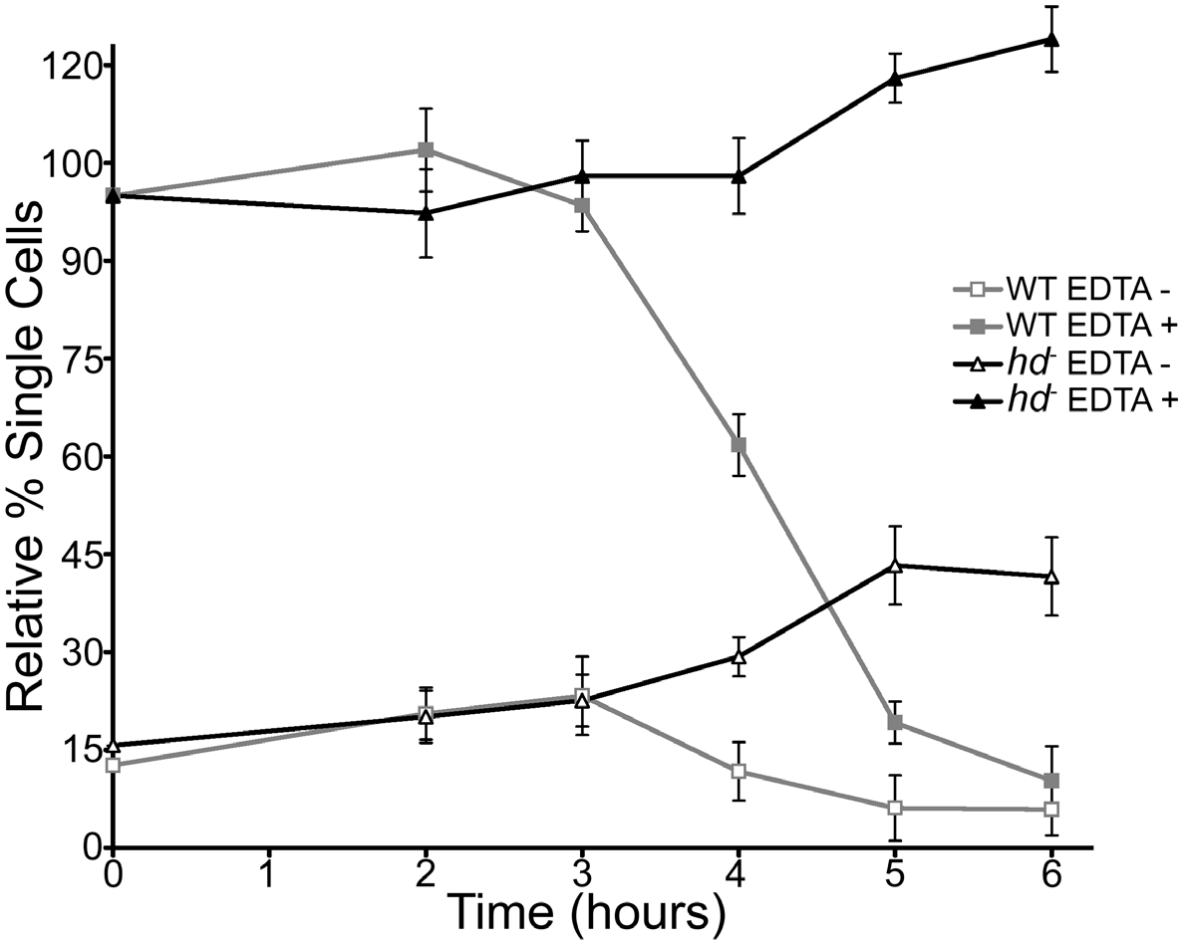
Loss of EDTA-resistant adhesion in *hd* ^−^ cells. Wild-type and *hd* ^−^ cells (5×10^6^ cells/mL) were developed in Soerensen's buffer at 150 rpm and 21°C. Samples were collected at the start of the assay and at one hour time points over a period of 6 hours. For each collection point, cells were dissociated by vortexing and then incubated in the presence or absence of 10 mM EDTA for 30 minutes, fixed with 2% glutaraldehyde (10 min.) and single cells were counted in triplicate using a hemocytometer. All experiments were performed in duplicate at least three times and the mean value for single cells in duplicate samples, expressed as percentage of total cells was plotted over time.

The cell-cell adhesion properties of *hd* ^−^ cells in the absence or presence of 10 mM ethylenediaminetetraacetic acid (EDTA) mirrored those observed for wild-type cells during the first 3 hours of development (Figure 7). However, after ∼4 hrs of development, wild-type cells began to attain EDTA-resistant cell-cell contacts whereas *hd* ^−^ cells not only failed to acquire EDTA-resistant cell-cell contacts but also began to display an overall loss in cell-cell cohesion as judged by an increase in the number of single cells assayed in the absence of EDTA (Figure 7). Even by 24 hours, neither EDTA-resistant adhesion in *hd* ^−^ cells nor recovery of their cohesion properties in suspension developed (data not shown). Thus, stringent conditions exacerbated *hd* ^−^ cell developmental deficits, revealing aberrant cell polarization, aggregation and motility consistent with abnormal cell shape, cytoskeletal function and cell-cell adhesion properties.

### *hd* ^−^ mutants exhibited abnormal prespore/spore cell differentiation

**Figure 8:**
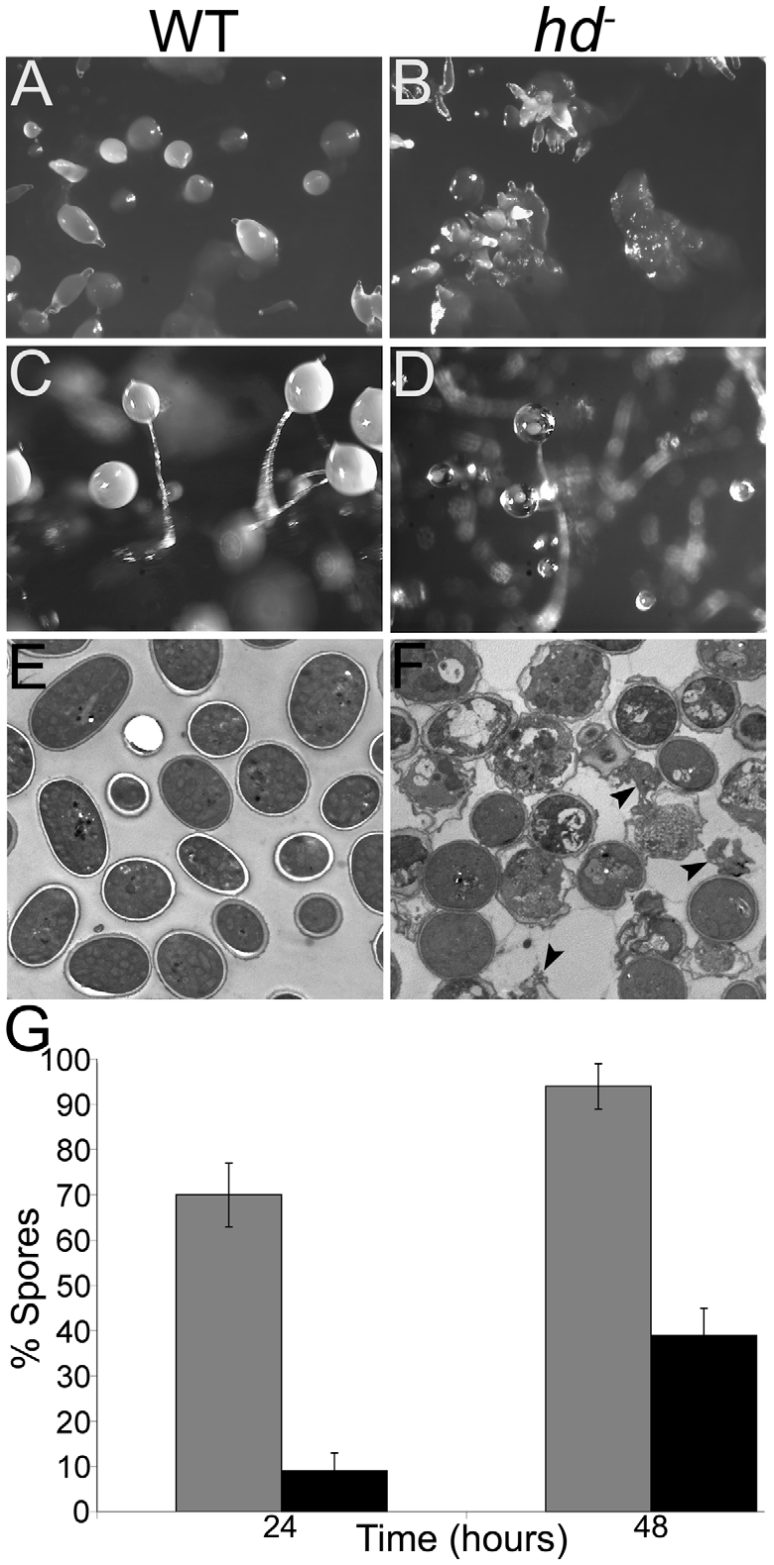
*hd* ^−^ cells formed mounds with multiple prestalk-tips and fruiting bodies with a glassy sorus. (A) Wild-type mounds formed mounds that develop a single tip which proceeded to form an elongated finger structure. (B) Multiple prestalk-tips formed atop most *hd* ^−^ mounds that went on to form comparably small individual finger structures. Cells were developed on SM agar with a lawn of *Klebsiella*. Images are top view. (C) Terminal structures of wild-type (*left panel*) and (D) *hd* ^−^ null cells (*right panel*). After 36–48 hours, the sori of *hd* ^−^ cells became progressively glassy in appearance and contained very few spores. (E) *hd* ^−^ spores spontaneously germinated within the sorus. Wild-type and *hd* ^−^ spores were collected and fixed (see Materials and Methods) for imaging using electron microscopy. Wild-type sori contained intact elliptical dormant spores with well-defined spore coats and without the presence of amoebae. (F) Spores collected from *hd* ^−^ fruiting bodies showed a heterogeneous mixture of round spores that represented the various stages of germination. Swollen spores, swollen spores with thin spore walls about to release amoebae, and nascent amoebae (*arrows*) are shown. (G) *hd* ^−^ mutants displayed defects in prespore/spore cell differentiation. Wild-type and *hd* ^−^ cells were starved at low density in monolayer culture in the presence of 15 mM 8-Br-cAMP to induce sporulation, viewed by brightfield microscopy and the number of spores formed was counted. The percentage of differentiated wild-type (*grey bars*) and *hd* ^−^ (*black bars*) spore cells was calculated from the total cells after 24 and 48 hour incubation periods. On average, *hd* ^−^ cells collectively formed spore cells at ∼30% efficiency relative to wild-type controls after 48 hours. Bars indicate standard errors that are derived from three independent experiments, each with three replicates.

Though streaming and aggregation of *hd* ^−^ cells were abnormal and overall development was delayed, multiple prestalk tips formed atop most of the developing *hd* ^−^ mounds. Each of these tips elongated into a finger structure, suggesting the possibility of aberrant prestalk/prespore cell differentiation (Figure 8A, 8B), and eventually produced a fruiting body with a glassy sorus containing a reduced spore complement when developed on KK_2_ agar or with a lawn of *Klebsiella* on SM agar (Figure 8C, 8D). In contrast to wild-type fruiting bodies, the numbers of spores in *hd* ^−^ sori decreased as the fruiting bodies aged, resulting in a glassy sorus; this suggested a potential defect in spore formation or alternatively, spore dormancy. We used electron microscopy to examine the fine structure of the elliptical wild-type spores and round mutant *hd* ^−^ spores from 72 hr fruiting bodies (Figure 8E, 8F). The EM images revealed the premature germination of spores in the sori of *hd* ^−^ fruiting bodies. In stark contrast to the dormant wild-type spores, the *hd* ^−^ sori contained a mixture of apparently dormant spores with three wall layers, swollen spores, swollen spores with thin spore walls about to release amoebae, and nascent amoebae (Figure 8F). Our data suggest that shortly after fruiting body formation *hd* ^−^ spores are unable to maintain dormancy, or alternatively, fail to achieve dormancy possibly through poor differentiation properties and germinate within the sorus. Consequently, we assessed the ability of *hd* ^−^ cells to differentiate into stalk and spore cells in low cell density monolayer culture [37]. In response to 10–100 nM of the stalk cell inducer, differentiation inducing factor 1 (DIF-1), *hd* ^−^ mutants were able to produce stalk cells as efficiently as wild-type cells (data not shown). However, in response to the sporulation inducer 8-Br-cAMP (5–15 mM), a cell permeable analogue of cAMP, sporulation in the mutants was delayed ∼24 hours compared to wild-type cells (Figure 8G). *hd* ^−^ cells collectively formed spore cells at ∼40% efficiency relative to wild-type controls after 48 hours differentiation (Figure 8G).

### *hd* ^−^ mutants produced spores with abnormal morphology and reduced viability

**Figure 9:**
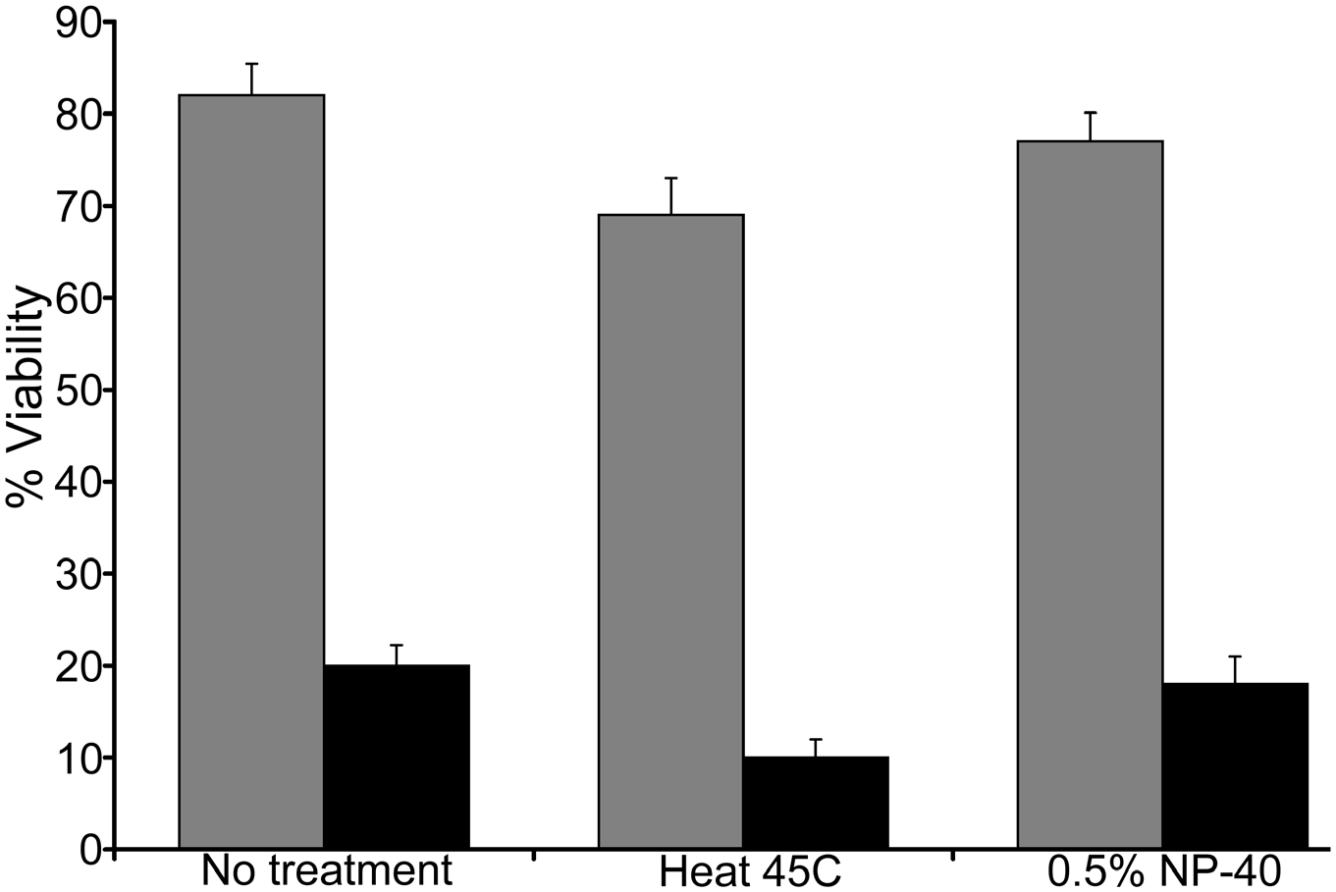
*hd* ^−^ cells produced spores with reduced viability. The viability of wild-type (*grey bars*) and *hd* ^−^ (*black bars*) spores was assessed. Spores were untreated, heated to 45°C for 10 minutes or incubated with 0.5% NP40 detergent for 5 minutes and aliquots of 100 spores were plated in triplicate onto SM-5 agar plates in a suspension of bacteria and grown for 7 days at 21°C. The relative viability of *hd* ^−^ spores was assessed by counting the number of clear plaques formed on the bacterial lawns. Results are representative of three independent experiments.

At least some spores produced by development of *hd* ^−^ organisms were capable of germination and formation of viable amoebae. The spore coats of both wild-type and *hd* ^−^ cells also stained brightly with Calcofluor, demonstrating the normal presence of cellulose in the mutant spores (data not shown). To test the relative viability of the *hd* ^−^ mutant spores, we compared their germination rates with wild-type spores. Spores harvested from sori of each cell line were mixed with *Klebsiella*, deposited on SM agar plates and the number of plaques that formed was scored. Spores harvested from wild-type sori displayed an average survival rate of 82±3.4%, whereas *hd* ^−^ spores demonstrated a dramatically reduced mean survival rate of 20±2.2% (Figure 9). This did not appear to be due to reduced resilience of *hd* ^−^ mutant spore coats, as spore viability of both wild-type and *hd* ^−^ mutant spores was not differently altered by treatment with the non-ionic detergent NP-40 (0.5%) and brief heat treatment (45°C) (Figure 9).

### Developmental defects in huntingtin-deficient organisms are cell-autonomous

**Figure 10:**
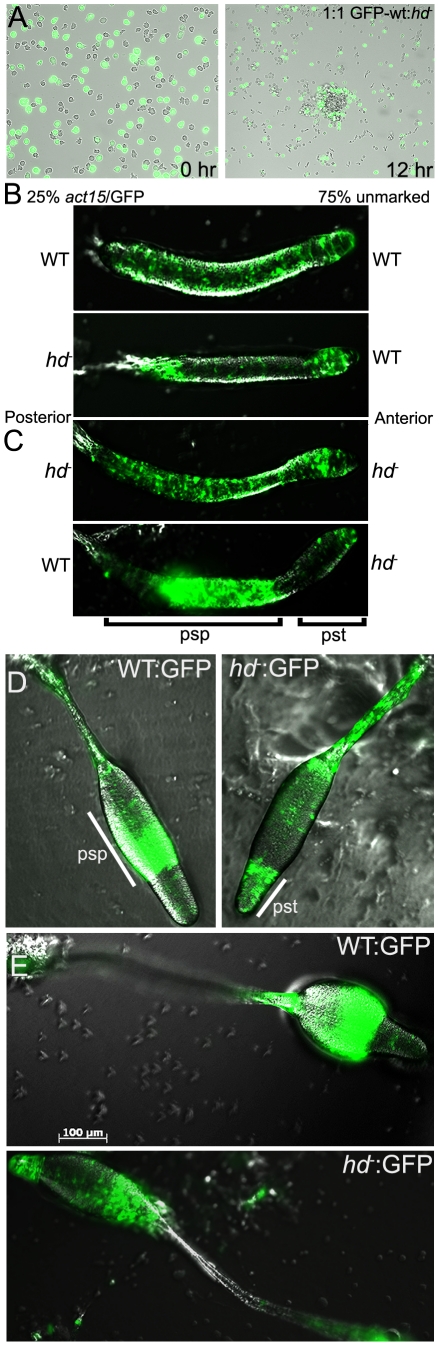
Huntingtin regulates prespore/spore differentiation cell-autonomously. GFP was expressed in wild-type cells and their position in chimeric aggregation territories with unlabelled *hd* ^−^ cells was monitored. (A) Mixed (1∶1) cell suspensions were submerged (1×10^5^ cells/cm^2^) under KK_2_ buffer (left panel). After 12 hours, aggregation centers were imaged under brightfield and fluorescence using an inverted microscope. *hd* ^−^ cells failed to populate the central region of aggregation territories but were instead localized to the periphery of the mound. Images are representative of three independent experiments. (B) Wild-type and *hd* ^−^ cells that express GFP were mixed 1∶3 or less with unmarked wild-type cells as indicated and cellular organization in live cells during the slug stage was assessed. GFP-labeled wild-type cells were mixed with the same genotype as a control (*top panel*); GFP-labeled *hd* ^−^ cells populated the prestalk regions of the slug (*lower panel*). (C) GFP-labeled *hd* ^−^ cells were mixed with the same genotype as a control (*top panel*); GFP-labeled wild-type cells populated the prespore regions of the slug (*lower panel*). Slugs are positioned with their indicated posterior prespore (psp) zones to the left and anterior prestalk (pst) zones to the right. (D) *hd* ^−^ cells marked with an actin/GFP reporter plasmid were mixed with unmarked wild-type cells in a 1∶3 ratio and allowed to develop into fruiting bodies. Developing structures (late culminant) were imaged by DIC and fluorescent microscopy. Wild-type cells marked with GFP (*left panel*) predominantly occupied the prespore/spore region; *hd* ^−^ cells marked with GFP failed to populate the prespore region and were overrepresented in the upper cup, lower cup and basal disc (*right panel*). Psp – *prespore*; Pst – *prestalk*. (E) Terminal fruiting structures were viewed by DIC and fluorescent microscopy. Wild-type cells expressing GFP (*top panel*); *hd* ^−^ cells expressing GFP (*lower panel*). Results are representative of three independent experiments for all panels shown. Scale bar 100 µm.

To distinguish whether the developmental defects observed in *hd* ^−^ cells were cell autonomous or non-cell autonomous, we followed the cell fate of *hd* ^−^ cells during chimera development in mixed cell cultures. Mutant *hd* ^−^ cells and wild-type cells carrying an act15/GFP construct, which marks all cells, were prepared and mixed in ratios of 1∶1, 1∶3 or 1∶9 with unmarked cells of the opposite genotype (or of the same genotype as controls). As expected, the deficits revealed by *hd* ^−^ cells in assays at the single cell stage (e.g., cell rounding, actin cytoskeleton rearrangement, sensitivity to osmotic shock) or early in development (cell-cell adhesion) were not rescued by the presence of wild-type cells in co-culture (data not shown). Similarly, in developing co-aggregates of cells developed under starvation buffer, *hd* ^−^ cells failed to populate the central region of the developing aggregate, consistent with the inability of wild-type cells to correct the observed failure in the initiation of intracellular cAMP signaling pathways required to trigger aggregation (Figure 10A) or, possibly, the onset of cell-cell adhesion properties in *hd* ^−^ cells.

We then studied cell pattern organization of live cells during the slug stage, where prestalk cells primarily populate the anterior region and prespore cells occupy the slug posterior [38]. In the context of an excess of unmarked wild-type cells (3∶1 ratio), GFP-marked *hd* ^−^ cells within chimeric slugs were distributed within the prestalk zone (pstO), rearguard and anterior-like cells (ALC), which suggests that they have a greatly reduced ability to form prespore cells in the presence of wild-type cells (Figure 10B). In the complementary experiment, GFP-marked wild-type cells co-developed with *hd* ^−^ cells populated primarily the prespore region at cell ratios of 1∶1 or lower (Figure 10C). In both comparable control experiments, GFP fluorescence was observed throughout the entire slug. Furthermore, this trend was also seen in terminally differentiated chimeric fruiting bodies with wild-type cells predominantly found in the spore mass whereas *hd* ^−^ cells occupied the tip and lower cup region, stalk and basal disc (Figure 10D, 10E). Thus, although *hd* ^−^ cells were capable of forming spores when developed as a pure population (albeit poorly, with reduced viability), the presence of wild-type cells in a chimeric organism did not rescue but rather reduced the contribution of *hd* ^−^ cells to spore formation.

## Discussion

Huntingtin is notable for its role in HD, where an expanded polyglutamine tract near the amino terminus of human huntingtin produces late-onset progressive neurodegeneration, likely through a modulatory effect of the polyglutamine region on the structure and/or function of the protein. In this study, we have investigated the fundamental function(s) of huntingtin in the haploid eukaryote *Dictyostelium discoideum*, by characterizing deficiency phenotypes in the single and multicellular phases of development of this social amoeba. The deletion of the *hd* gene, using targeted homologous recombination, was compatible with cell growth but produced pleiotropic cell autonomous phenotypes that demonstrated that mutant cells could not efficiently complete the processes necessary for coordinated synchronous development of a multicellular organism. The pleiotropic effects of huntingtin deficiency could be the result of loss of a single activity or could be separate effects due to the protein having multiple functions. Further experimentation will be required to resolve these options, but the multiple phenotypes, at both single cell and multicellular stages, offer several routes to further explore the issue.

Our findings suggest that huntingtin participates, directly or indirectly, in actin cytoskeleton-membrane dynamics that affect cell shape. The mechanical properties of the cytoplasm are important determinants of cell shape and permit cells to change the cytoplasm from a rigid to a dynamic actin network that can greatly influence cell motility [39]. Huntingtin under certain conditions appears to act as an essential facilitator of this process. Whereas *hd* ^−^ cells displayed apparently normal cell shape, pseudopod formation, F-actin localization and random motility in nutrient-rich media, when assayed in the absence of nutrients (under developmental buffer), they failed to produce membrane extensions, were abnormally round and lacked cortical F-actin, although at high cell density, rounded *hd* ^−^ cells managed to form delayed aggregation territories by accretion. These observations could also reflect the loss of an excitatory signaling pathway during development of *hd* ^−^ cells, as the phenotypes resemble those exhibited by *Dictyostelium* null mutants for adenylyl cyclase that overexpress the catalytic subunit of protein kinase A (PKA) [40] or cells expressing the constitutively active inhibitory heterotrimeric G-protein Gα9 [41]. Addition of exogenous pulses of 75 nM cAMP at 6 minute intervals over a period of 4–5 hours did not rescue the aggregation defect in our *hd* ^−^ cells (data not shown), though it is plausible that high cell density partially rescues mound formation by increasing cell-cell contacts. However, cAMP signaling and subsequent chemotactic aggregation could be rescued through the addition of bivalent cations (Ca^2+^ or Mg^2+^) to the medium, which suggests the possibility that *hd* ^−^ cells exhibit depleted cationic stores. Our findings support the notion that huntingtin may facilitate proper Ca^2+^- Mg^2+^-dependent actin cytoskeleton remodeling that determines cell shape, but that the detection of this role critically depends upon the conditions in which the cells are evaluated.

A second function for huntingtin is maintenance of cellular integrity under conditions of osmotic stress. Contractile vacuoles are intracellular membrane organelles involved in osmoregulation, function as a highly efficient acidic Ca^2+^-store that is required for cAMP-induced Ca^2+^-influx and are found in free-living amoebae and protozoa [42]–[44]. Normally, wild-type cells growing in culture media contain a few moderately active contractile vacuoles that maintain the osmotic balance of the cell. The contractile vacuole system functions as a “bladder” during conditions of hypoosmotic stress, where it collects fluid (water and neutral amino acids) in a network of tubular channels that associate with the cortical actin network to allow for transient fusion with the plasma membrane and expulsion of its contents to the extracellular environment [45], [46]. The behavior and survival rate of *hd* ^−^ cells in response to hyperosmotic conditions is similar to wild-type cells (data not shown). However, contractile vacuole activity in response to hypoosmotic stress is completely abolished in *hd* ^−^ cells, and renders the cells sensitive to conditions of low ionic strength. These effects were consistently associated with a retraction of F-actin from the cell cortex to the cytosol, suggesting that in the absence of huntingtin, inefficient actin cytoskeleton remodeling may underlie the failure of the tubular network to convert into the contractile vacuole. This could involve abnormal formation of the vacuole and failure to discharge, or altered regulation of F-actin binding proteins, as there are examples of both. Cells deficient for MEGAP1 and MEGAP2, members of the Pombe/Cdc15 homology (PCH) family of proteins involved in actin cytoskeletal reorganization, are sensitive to hypoosmotic conditions because they are defective in the tubulation and the associated emptying of contractile vacuoles [47]. Whereas MEGAP-null cells fail to form tubules from vacuoles and therefore accumulate the latter, we suggest that *hd* ^−^ cells cannot effectively osmoregulate due to the inability of the tubule system to convert to vacuoles. Indeed, *Dictyostelium* double mutants lacking two F-actin crosslinking proteins, α-actinin and gelation factor, display a general weakening of the cortical cytoskeleton and, like *hd* ^−^ cells, do not exhibit the normal polarized morphology of wild-type cells during aggregation and are sensitive to hypoosmotic shock [48]. Our data further suggest that the defective CV system in *hd* ^−^ cells renders cells not only sensitive to extreme hypoosmotic shock, but secondarily affects intracellular ion homeostasis as the CV has been shown to function as a major Ca^2+^-store [42], [44] as well as being tightly linked to Ca^2+^/Mg^2+^-containing mass dense granules [43]. As a consequence, unless these cations are provided exogenously *hd* ^−^ cells appear unable to initiate cAMP-induced Ca^2+^-transients that may act in a feedback loop to positively reinforce cAMP relay [44] leading to chemotaxis. Moreover, in contrast to wild type cells, development of *hd* ^−^ cells on non-nutrient agar was blocked by EGTA and, interestingly, was not differentially sensitive to low concentrations of caffeine (data not shown) suggesting that a defect in cAMP signaling, if present, is relatively minor in comparison to their sensitivity to low osmolarity and/or cation chelation. We posit that huntingtin likely acts very early in establishing CV activity or integrity under hypoosmotic conditions. It may act indirectly, or it may function directly as a regulatory scaffold in this setting to drive assembly of the cortical cytoskeleton with CV-associated proteins during the conversion of tubule to vacuoles. Importantly, several proteins associated with the contractile vacuole network and involved in membrane protein trafficking are related to human proteins, suggesting that some functions of the contractile vacuole network have been preserved during the evolution of higher eukaryotes [45], [49].

The cell-cell adhesion properties of *hd* ^−^ cells are similar to wild-type cells during the first 3 hours of development, but as development proceeds *hd* ^−^ cells fail to acquire EDTA-resistant contacts. This suggests that *hd* ^−^ cells retain the ability to form early functional adhesion sites but not the EDTA-resistant sites characteristic of aggregating chemotactic cells. Cell-cell contact plays an important role in differentiation and gene expression in *Dictyostelium* [50]–[52]. EDTA-resistant cell-cell binding is mediated by the expression of glycoprotein gp80 at the onset of aggregation [53], [54]. Interestingly, the expression of gp80 is greatly augmented by pulsatile cAMP signaling and the formation of EDTA-sensitive cell-cell contacts [51], [55]. Taken together, the apparently normal EDTA-sensitive adhesion properties of *hd* ^−^ cells, their inability to initiate signaling centers at densities where wild-type cells can, and the rescue of cAMP relay and streaming by the addition of exogenous cations suggest that *hd* ^−^ cells potentially lack the Ca^2+^- Mg^2+^-dependent excitatory intracellular signal transduction pathways that upregulate gp80 EDTA-resistant adhesion complexes, or that they are deficient in transporting gp80 to the cell surface.

We also provide compelling evidence to suggest huntingtin modulates prespore cell fate choice. In the context of either an equal ratio or excess ratio (3∶1 and 9∶1) of unmarked wild-type cells, GFP-marked *hd* ^−^ cells within chimeric slugs are distributed within the prestalk zone (pstO), and as rearguard and anterior-like cells (ALC) which suggests they have a significantly reduced ability to form prespore cells when challenged in the presence of wild-type cells. This could be due to a defect in the mutant cells in cell-cell interaction, either through extracellular signaling or direct contact relative to the efficient homotypic interactions of wild-type cells. Indeed, this difference in differentiation might be explained by the adhesion defect in *hd* ^−^ cells as, in chimeras with wild-type, gp80-null cells are also directed preferentially toward the prestalk pathway [56]. Differential differentiation of huntingtin deficient cells during development has also been observed in vertebrates. In zebrafish, reduced expression of huntingtin differentially targets development of telencephalic neurons compared to mid- and hind-brain [57]. In mouse chimeras, *Hdh* ^−/−^ cells also preferentially colonize the hypothalamus, midbrain, and hindbrain during relative to the telencephalon and the thalamus during early development [58]. Thus, like these latter neuronal populations, *Dictyostelium* cells require huntingtin for the proper development of viable spores in the presence of wild-type cells.

The culmination of *Dictyostelium* multicellular development permits survival of the organism through the production of environmentally resistant spores and the complex organization of actin filaments in *Dictyostelium* spores contributes to their ellipsoid shape and dormancy [59]. *hd* ^−^ mutants produced abnormally round spores, which exhibited decreased viability, suggesting that they were poorly differentiated or had a defect in the actin-cytoskeleton. Moreover, electron microscopic examination of the fine structure of spores from wild-type and *hd* ^−^ fruiting bodies suggested that the formation of the glassy sorus was likely a result of the premature germination of spores and the resulting death of newly emerged amoebae. Spore dormancy is maintained via active biological processes including high osmolarity, actin dynamics, production of the germination inhibitor discadenine and active PKA which in concert function to insure a viable supply of spores [60]–[62]. Since *hd* ^−^ amoebae are sensitive to changes in osmolality, display aberrant F-actin staining and show compromised sporulation when subjected to constitutive PKA activation *via* 8-Br-cAMP *in vitro*, it is plausible that in the absence of huntingtin, imprecise cytoskeletal architecture and signaling from PKA required to maintain or enter dormancy might also be defective at this late stage of development.

Our data do not provide a simple definition for a single normal function for huntingtin, but, as huntingtin deficiency in *Dictyostelium* produces pleiotropic effects throughout the life cycle, our findings are consistent with the consensus from mammalian studies that huntingtin is a multifunctional protein that can impact upon many biochemical processes. In *Dictyostelium*, defects in CV activity or integrity leading to a disruption in ion homeostasis affecting the cytoskeleton, cell shape and cell-cell adhesion would all predictably interfere with many aspects of chemotactic aggregation and development. Defining the degree to which the phenotypes reported here are connected by common underlying biochemical deficits or, alternately, reflect different functions of huntingtin will require detailed molecular investigation. However, the existence of a *Dictyostelium* ortholog of human huntingtin, the viability of the null *hd^−^* mutant, and its discrete, readily assayed deficiency phenotypes indicate that this haploid organism provides an effective genetic model system to identify molecular and cellular processes affected by the loss of huntingtin function. While the latter is of fundamental biological interest considering the unique nature of this ancient large *α*-solenoid HEAT protein, delineating which of these functions are conserved in mammals and determining whether they are altered by expansion of the polyglutamine tract in human huntingtin will also provide much needed insights into the mechanism by which mutant huntingtin triggers HD pathogenesis.

## Materials and Methods

### Cell culture and development

Wild-type *Dictyostelium discoideum* AX3 cells were grown in association with *Klebsiella aerogenes* on SM plates, axenically in tissue culture plates or as shaking cultures in HL-5 medium (Formedia) at 21°C. To assess growth rates, shaking cultures (150 rpm) of AX3 or *hd^−^* null mutant cells were suspended in HL-5 at a density of 1×10^4^ cells/mL and grown at 21°C. Cells were counted in triplicate using a hemocytometer. For synchronous development exponentially growing cells (∼2×10^6^ cells/mL) were washed three times from the nutrient source with DB buffer (5 mM Na_2_HPO_4_, 5 mM KH_2_PO_4_, 1 mM CaCl_2_, 2 mM MgCl_2_, pH 6.5) by centrifugation at 1500 rpm for 5 min, resuspended at a density of 5×10^7^ cells/mL, deposited on black nitrocellulose filters supported by filter pads soaked in DB buffer and developed at 21°C. Growth curves were determined for the *hd* ^−^ strain, and parental (wild-type) strain AX3. Shaking (150 rpm) cultures were grown axenically in HL-5 at 21°C. Exponentially growing cultures were used to inoculate four 50 mL cultures for each strain, starting at 1×10^5^ cells per mL and counted daily using a hemocytometer. For development under buffer (KK_2_), cells were grown in 6-well tissue culture dishes in HL-5 to a density of ∼5×10^5^ cells/cm^2^. Cells were washed twice with KK_2_ and then allowed to develop under KK_2_ or KK_2_ supplemented with 1 mM CaCl_2_, 1 mM MgCl_2_, 1 mM MgSO_4_ or were provided with 75 nM pulses of cAMP every 6 minutes for a period of 4 hours [41]. For development and assessment of streaming behavior cells were washed twice with KK_2_ and then deposited on agar at a density of ∼5×10^5^ cells/cm^2^ on non-nutrient 1.5% agar plates (KK_2_ buffer pH 6.4) or KK_2_ supplemented with caffeine (1, 2.5 and 5 mM) or 1 mM EGTA.

### Disruption of the *hd* gene by homologous recombination

The targeting construct for disruption of the *hd* gene was made by standard cloning procedures in the floxed-Bsr gene disruption vector pLPBLP [63]. PCR was used to amplify genomic sequences flanking and within the coding region of the *hd* gene. The 5′ targeting region of *hd* was amplified using primers 5′CCCGGTACCATGGATCTTATTCG3′ and 5′-CCCAAGCTTCCAATGATAATATA3′ which incorporate restriction sites (underlined) for *Kpn*I and *Hind*III, respectively to facilitate cloning into the vector pLPBLP. The 3′ targeting region of the *hd* gene was amplified using primers 5′CCCCTGCAGTTCTCCACCAATCT3′ and 5′CCCGGATCCGTTATATGATCGG3′ which incorporate restriction sites (underlined) for *Pst*I and *BamH*I, respectively to facilitate directional cloning into pLPBLP. For electroporation, 5×10^6^ cells in 100 µL of ice cold buffer H-50 (20 mM HEPES, 50 mM KCl, 10 mM NaCl, 1 mM MgSO_4_, 5 mM NaHCO_3_, 1 mM NaH_2_PO_4_) was mixed with 10 µg of linearized gene-targeting DNA and electroporated twice, waiting for about 5 sec between pulses, using a Bio-Rad Gene Pulser (0.85 kv, 25 µF) (Bio-Rad, Hercules, CA) [64]. The next day, the media was replaced with fresh HL-5 supplemented with 10 µg/ml blasticidin. Single colonies were collected and replica-plated into multiple 96-well plates. Genomic DNA was extracted exactly as described [65], and targeted gene disruptions were identified initially by several PCR reactions using a combination of primers (Table S1). Clonal isolates from multiple independent mutants possessing the same disruption and sporulation phenotype were obtained. One of these clones (httE13) was used in all subsequent experiments. Southern blot hybridizations using Bsr DIG-labeled probes were used for definitive confirmation of gene disruption. The *Bsr* fragment from pLPBLP was agarose gel purified and used to make a DIG-labeled probe using the DIG-High Prime starter kit from Roche. Hybridization was carried essentially as described in the DIG-DNA Detection manual (Roche). Blots were washed at room temperature and then at 68°C, equilibrated in detection buffer, incubated in disodium 3-(4-methoxyspiro {l,2-dioxetane-3,2′-(5′-chloro)tricyclo[3.3.1.1]decan}-4-yl) phenyl phosphate (CSPD) for 15 minutes at 37°C and developed using enhanced chemiluminescence XOMAT Kodak film.

### RT-PCR analysis of *hd* splice variants

The analysis of total RNA for *hd* alternate splice variants was performed using exon-specific primers and RT-PCR. Total RNA was collected from vegetative cells and at 6-hour increments during development using the Qiagen RNeasy kit followed by on-column DNase digestion (Qiagen). The *hd* transcript was detected using the Qiagen One-Step RT-PCR kit as per the manufacturer's recommendations using primer pairs listed in Table S2.

### Conventional and fluorescence microscopy

Growing cells were harvested and deposited into Lab-tek chambered cover glass (8 well) at 1×10^5^ cells/cm^2^ and allowed to grow overnight in HL-5 at 21°C. For F-actin staining, the media was aspirated and cells were fixed with 4% formaldehyde in PDF buffer (20 mM KCl, 11 mM K_2_HPO_4_, 13.2 mM KH_2_PO_4_, 1 mM CaCl_2_, 2.5 mM MgSO_4_, pH 6.4) at room temperature for 15 minutes, permeabilized with 0.025% TX-100 in PDF buffer for 15 minutes, followed by a brief wash in PDF buffer. Cellular F-actin was stained by incubating the fixed cells with Texas-Red-conjugated phalloidin (Molecular Probes, Eugene, OR) at a concentration suggested by the manufacturer for 25 minutes at room temperature and washed three times with PBS prior to viewing. To assess the effects of nutrient removal, the HL-5 was aspirated and replaced with KK_2_ buffer (16.5 mM KH_2_PO_4_ and 3.8 mM K_2_HPO_4_). After starving cells for 1 hr, the cells were fixed and processed for F-actin staining as described above. Nuclei were stained with Hoechst 33342. Images were taken on an inverted NIKON microscope TE2000 (NIKON Instruments, Dallas, TX) with either 4×, 20×, 40× objectives or a 63× 1.4 NA PlanFluor oil immersion objective and Quantix camera (Roper Scientific, AZ) controlled by NIS Elements and processed with Adobe Photoshop software (Adobe, San Jose, CA).

### Spore viability assays

The viability of wild-type and *hd* ^−^ spores were assayed by harvesting approximately 2×10^8^ cells and plating onto 10 cm non-nutrient 1.5% agar plates (KK_2_ buffer pH 6.4). Spores from mature fruiting bodies were harvested using sterile pipette tips containing 10 µL of spore buffer (40 mM KH_2_PO_4_, 20 mM KCl, 2.5 mM MgCl_2_), washed twice by centrifugation at 12,500 g for 2 minutes at room temperature and counted with a hemocytometer. Aliquots of 100 spores were heated to 45°C for 10 minutes, incubated with 0.5% NP40 detergent (Sigma-Aldrich, St Louis, MO) for 5 minutes or incubated with spore buffer alone for 5 minutes as a control. Spores were washed and plated in triplicate onto SM-5 agar plates in a suspension of bacteria (either *E. coli* B/R or *K. aerogenes*) and grown for 7 days at 21°C. The viability of spores was assessed by counting the number of clear plaques formed on the bacterial lawns for each treatment. Relative viability was measured as the percentage of *hd* ^−^ plaques formed compared to wild-type cells for all conditions.

### Submerged-monolayer assay

The submerged-monolayer assay was modified from an assay described previously [66]. For stalk cell induction, vegetative cells were washed once with KK_2_ buffer and three times with stalk buffer (10 mM morpholineethanesulfonic acid [MES], 2 mM NaCl, 10 mM KCl, 1 mM CaCl_2_, 5 mM cAMP and 200 µg of penicillin-streptomycin per ml, pH 6.2). The cells were then plated into 24-well tissue culture plates at a density of 10^4^ cells/cm^2^ and incubated in the presence or absence of 10–100 nM differentiation-inducing factor (DIF-1). A calcofluor solution (0.01%) was added to the wells for 5 min., removed and the cells were observed immediately by microscopy. Only the cells that were vacuolated and stained by calcofluor were counted as stalk cells [67]. For spore cell induction, wild-type cells and *hd* ^−^ cells were washed once with KK_2_ buffer and three times with spore buffer (10 mM MES, 20 mM NaCl, 20 mM KCl, 1 mM CaCl_2_, 1 mM MgCl_2_) and then incubated in spore buffer supplemented with 5–15 mM 8-Br-cAMP for 24 and 48 hours [68] and visualized in bright field microscopy. Relative sporulation percentage was calculated as described for the stalk cell assay. Assays were performed three independent times, each with three replicates.

### Development of chimeric mixture of GFP-labeled wild-type and *hd* ^−^ cells

In *Dictyostelium*, chimeras are readily produced by mixing cells of different genetic backgrounds and allowing them to co-aggregate to form a chimeric mound [69]. For chimera aggregation, development and tracing cell lineages, wild-type and *hd* ^−^ cells were transfected with pTX-GFP and selected in 10 µg/mL G418. GFP-marked and unmarked cells were mixed at varying percentages prior to deposition under buffer or development on non-nutrient agar as described above.

### Electron microscopy (EM)

To examine spores under electron microscopy, greater than 2×10^8^ wild-type and huntingtin-null spores were harvested and resuspended in 2% glutaraldehyde (EM grade) in spore buffer and incubated for 1–2 hours at 21°C. After this fixation, the spores were washed in spore buffer and processed for electron microscopy (EM). Electron micrographs were prepared using the CHGR Microscopy Core.

### Generation of DdHtt (595):GFP reporter construct

*Dictyostelium* expression vector pTX-GFP was digested with restriction enzymes *Sal*I and *Kpn*I, releasing its actin15 promoter and the first 11 codons of the ORF containing an 8× His tag [70]. A region of DNA sequence including 595 bp 5′ to *hd* ORF and the first 42 bp of the ORF was amplified from *D. discoideum* (AX3) genomic DNA using forward primer DdHtt_F608+SalI (5′- AATATGTGTCGACCTACAGTTATTAAATAAATTGCAATAAAGGTGC-3′) and reverse primer DdHtt_R_Pro+*Kpn*I (5′-TCCTCTGTGGTACCTGGTGATGCTGATAATATATCTAATCCACG-3′). The product was digested with restriction enzymes *Sal*I and *Kpn*I to facilitate ligation into the vector upstream and in frame with the GFP ORF sequence. The resulting vector therefore expresses the first 14 amino acids of *hd* fused upstream of GFP, under the control of the predicted minimal *hd* promoter sequence. The vector was transformed into AX3 cells as previously described [71].

### Cell–cell adhesion assay

Cell-cell adhesion was assessed as previously described [72]. Briefly, cells were grown axenically to a density of 2–5×10^6^ cells/mL, washed twice in ½ volume of ice cold Soerensen buffer (SB), resuspended in 0.4 initial volume of SB and vortexed, then immediately counted in order to adjust the concentration of cells to 5×10^6^ cells/mL. Cells were incubated in an Erlenmeyer flask at 150 rpm and 21°C and samples were collected at various time points over a period of 6 hours. For each collection point, cells were incubated in the presence or absence of 10 mM EDTA for 30 minutes and fixed with 2% glutaraldehyde. Single cells were counted using a hemocytometer. All experiments were performed in duplicate and the mean value for single cells in duplicate samples, expressed as percentage of total cells was plotted over time.

### Osmotic shock

Control (wild-type) or *hd* ^−^ cells were grown HL-5 and the medium was replaced with low ionic buffer (KK_2_), water (hypotonic) or 400 mM sorbitol in KK_2_ (hyperosmotic). At various time points samples were taken, diluted into KK_2_ phosphate buffer containing *K. aerogenes* and plated onto SM agar plates to assay for viability. Cell lysis upon osmotic shock was also observed visually by brightfield microscopy. All experiments were performed in triplicate.

## Supporting Information

Figure S1 Comparison of growth rates between wild-type and *hd* ^−^ cells. Cells were inoculated into fresh HL-5 medium at an initial density of 5×10^4^ cells/ml and incubated at 21°C with shaking at 150 rpm on an orbital shaker. Cell counts were performed every 24 hours, under a microscope, using a hemocytometer. The graph represents the average of the readings of the four flasks for each strain, at each time-point. *Hd* ^−^ cells grow with a doubling-time of ∼10 hours compared to the ∼12 hours doubling time of wild-type control cells in suspension culture.
(TIF)

Figure S2 The addition of bivalent cations to the medium rescues cAMP relay and streaming of *hd* ^−^ cells. (A) Wild-type and (B) *hd* ^−^ cells (1×10^5^ cells/cm^2^) were submerged under KK_2_ in the presence of 1 mM CaCl_2_ and allowed to develop for 6 hours. Long streams of wild-type cells are seen moving into aggregation centers (*left panel*). In the presence of Ca^2+^, *hd* ^−^ cells are now capable of streaming but form much smaller streams (*right panel*).
(TIF)

Table S1 Primers used for preliminary genomic DNA PCR screening of *hd-null* cells. Sequences of primers presented in the 5′-3′ direction that were used to initially identify putative *hd* ^−^ cells.
(DOCX)

Table S2 Primers used to detect the presence of alternatively spliced *hd* mRNA transcripts during growth and development. Sequences of primers presented in the 5′-3′ direction that was used in RT-PCR reactions to detect the presence of alternatively spliced *hd* transcripts from wild-type AX3 purified total RNA.
(DOC)

Video S1 Osmoregulation of wild-type *Dictyostelium* cells during hypoosmotic conditions. Growth media was replaced with water and cells were imaged using brightfield microscopy. After 2 hours in water, images were taken every 20 seconds over a period of 1 hour with a frame rate of 50 msec.
(AVI)

Video S2 Lysis of *Dictyostelium hd* ^−^ cells during hypoosmotic conditions. Growth media was replaced with water and cells were imaged using brightfield microscopy. After 2 hours in water, images were taken every 20 seconds over a period of 1 hour with a frame rate of 50 msec.
(AVI)

Video S3 Pulsatile cAMP signaling and chemotactic streaming of wild-type *Dictyostelium* cells. Pulsatile signaling of cAMP appears as dark, periodic propagating waves moving from right to left across the field of view prior to the onset of chemotactic streaming. Cells were imaged using brightfield microscopy and images were captured every 15 seconds over a period of 6 hours. Video is representative of the time between 5 and 6 hours with a frame rate of 50 msec.
(AVI)

Video S4 *Dictyostelium hd* ^−^ cells do not exhibit pulsatile cAMP signaling or chemotactic streaming into aggregation territories. *Hd* ^−^ cells fail to initiate pulsatile waves and consequently do not stream into aggregation centers. Cells can be seen randomly colliding to form loose aggregates. Cells were imaged using brightfield microscopy and images were captured every 15 seconds over a period of 8 hours. Video is representative of the time between 5 and 6 hours with a frame rate of 50 msec.
(AVI)

Video S5 Rescue of pulsatile cAMP signaling and chemotactic streaming of *Dictyostelium hd* ^−^ cells by addition of bivalent cations. Under KK_2_ buffer *Hd* ^−^ cells fail to initiate pulsatile waves and consequently do not stream into aggregation centers. However, addition of 1 mM CaCl_2_ (shown here) rescues pulsatile signaling and partially rescues chemotactic streaming. The streams formed by *hd* ^−^ cells under these conditions tend to break and are shorter than that seen with wild-type cells. Cells were imaged using brightfield microscopy and images were captured every 15 seconds over a period of 6 hours. Video is representative of the time between 5 and 6 hours with a frame rate of 50 msec.
(AVI)

Video S6 EGTA blocks calcium rescue of *hd* ^−^ early development. *Dictyostelium hd* ^−^ cells do not exhibit pulsatile cAMP signaling or chemotactic streaming into aggregation territories when incubated with 1 mM CaCl_2_ in the presence of 1 mM EGTA. *Hd* ^−^ cells failed to initiate pulsatile waves and consequently do not stream into aggregation centers, become rounded and can be seen randomly colliding. Cells were imaged using brightfield microscopy and images were captured every 15 seconds over a period of 8 hours. Video is representative of the time between 5 and 6 hours with a frame rate of 50 msec.
(AVI)
