# Supplementary figures and images for: Deficiency of Huntingtin Has Pleiotropic Effects in the Social Amoeba Dictyostelium discoideum
Source: PLoS Genet. 2011 Apr 28;7(4):e1002052. doi: 10.1371/journal.pgen.1002052 (PMC3084204; doi:10.1371/journal.pgen.1002052)

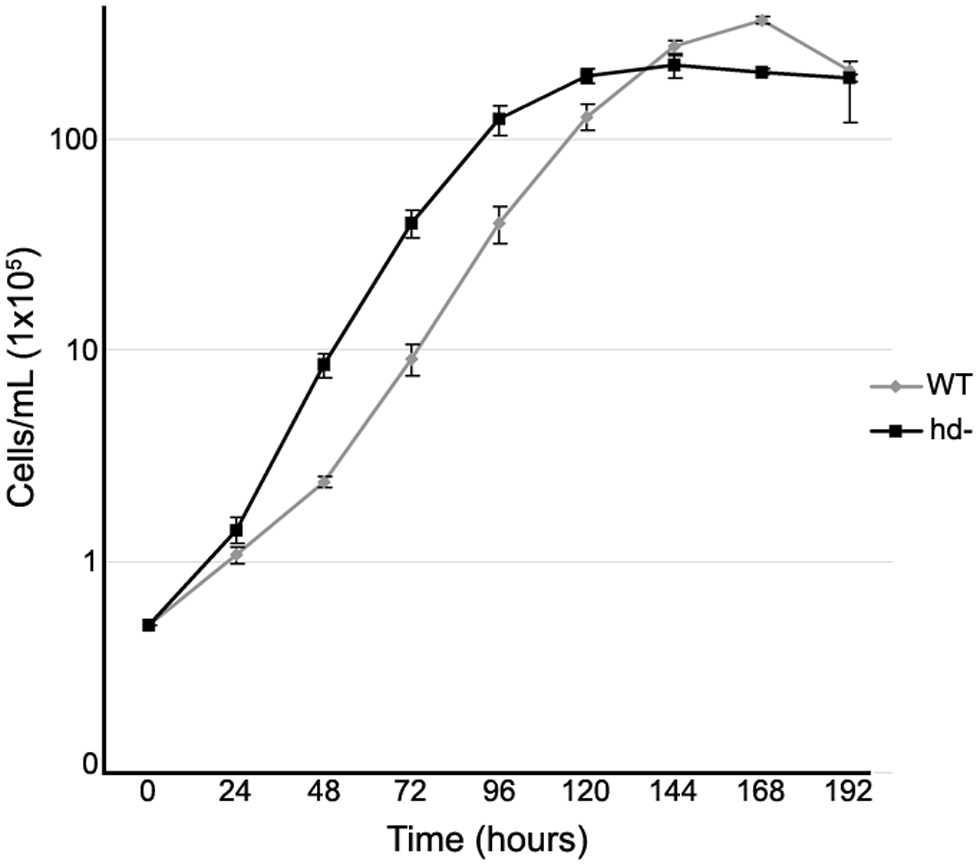

Supplement: Figure S1 — Comparison of growth rates between wild-type and hd − cells. Cells were inoculated into fresh HL-5 medium at an initial density of 5×10^4^ cells/ml and incubated at 21°C with shaking at 150 rpm on an orbital shaker. Cell counts were performed every 24 hours, under a microscope, using a hemocytometer. The graph represents the average of the readings of the four flasks for each strain, at each time-point. Hd − cells grow with a doubling-time of ∼10 hours compared to the ∼12 hours doubling time of wild-type control cells in suspension culture. (TIF) [file pgen.1002052.s001.tif]

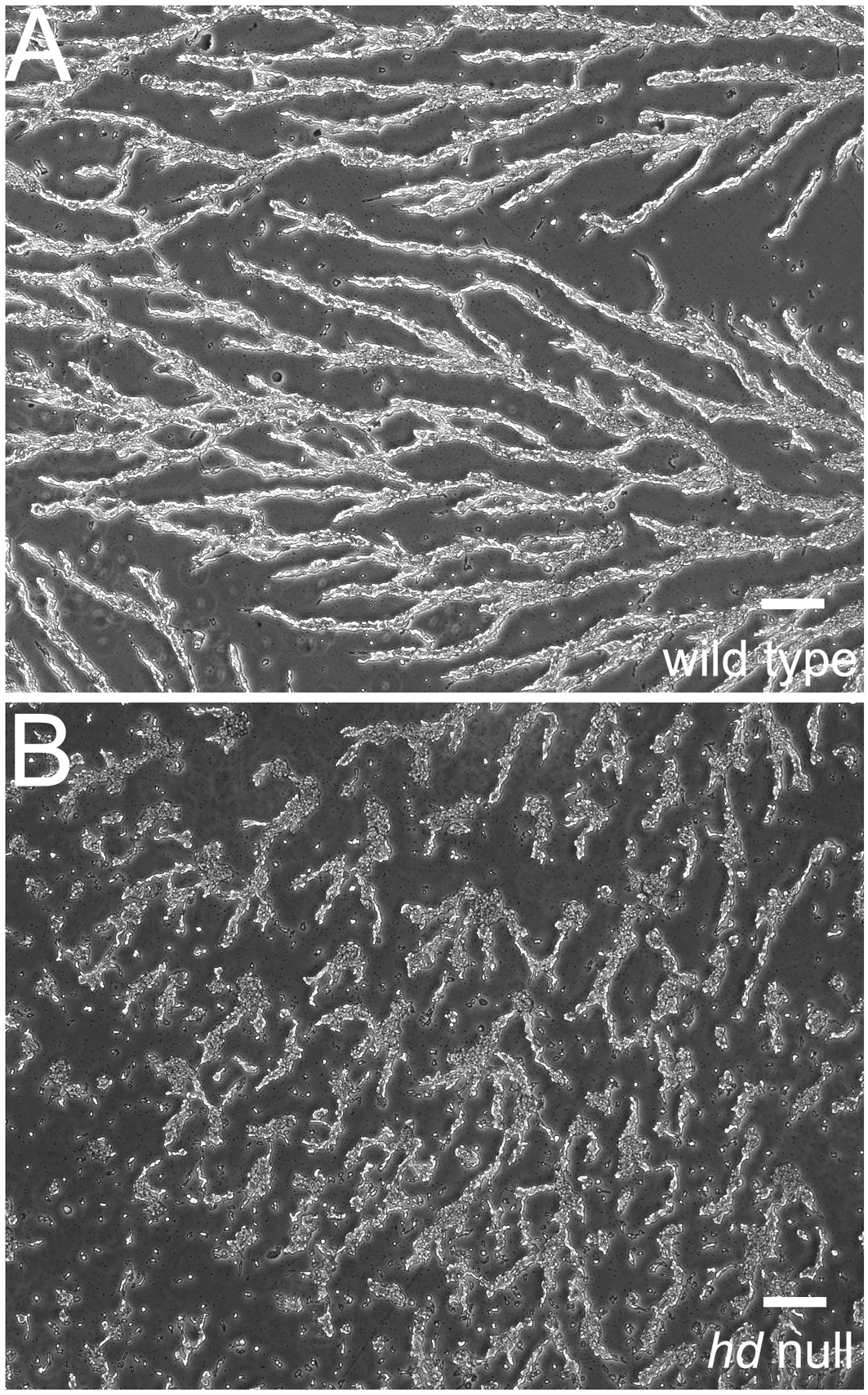

Supplement: Figure S2 — The addition of bivalent cations to the medium rescues cAMP relay and streaming of hd − cells. (A) Wild-type and (B) hd − cells (1×10^5^ cells/cm2) were submerged under KK2 in the presence of 1 mM CaCl2 and allowed to develop for 6 hours. Long streams of wild-type cells are seen moving into aggregation centers (left panel). In the presence of Ca2+, hd − cells are now capable of streaming but form much smaller streams (right panel). (TIF) [file pgen.1002052.s002.tif]
