# Supplementary material for: Deficiency of Huntingtin Has Pleiotropic Effects in the Social Amoeba Dictyostelium discoideum
Source: PLoS Genet. 2011 Apr 28;7(4):e1002052. doi: 10.1371/journal.pgen.1002052 (PMC3084204; doi:10.1371/journal.pgen.1002052)
Supplement: Table S1 — Primers used for preliminary genomic DNA PCR screening of hd-null cells. Sequences of primers presented in the 5′-3′ direction that were used to initially identify putative hd − cells. (DOCX) [file pgen.1002052.s003.docx]

**Table S1. Primers used for preliminary genomic DNA PCR screening of *hd-null* cells.**

| **Primer name** | **Primer Sequence (5’ – 3’)** |
| --- | --- |
| P1-DH_intFor | TATTGCATTGATAAAGTTTGGAGAG |
| P2-DH_intRev | TACTACTTGCAGGTATACATGATGC |
| P3-HtNF | ATGGATCTTATTCGTGGATTAGATATATTATC |
| P4-HKOinDR | TGAAATACTCTCCTGTAATGACTCATCC |
| P5-Dhtt_us63F | CGTATATAAAGCACACAATAATACACC |
| P5-Dhtt_us146F | AACACACACTCTCACACACCACAGC |
| P6-(act)OL1_R | TTCAAATAATAATTAACCAACCCAAG |
| P6-(act)OL2_R | TTTTCAAATAATAATTAACCAACCCA |
| P7-(act)OL3_F | TCAAAAAGATAAAGCTGACCCGAAAGC |
| P7-(act)OL4_F | GAAAATCAAAAAGATAAAGCTGACCCG |
| P8-Dhttr80_R | CGTGCATTAATGGATTCAAGTTCGAT |
| P8-Dhttr109_R | CTAATACCTGTACAAGCTAATTTCGC |
| rnlA_F | ttacatttattagacccgaaaccaagcg |
| rnlA_R | ttccctttagacctatggaccttagcg |
