# Supplementary material for: Deficiency of Huntingtin Has Pleiotropic Effects in the Social Amoeba Dictyostelium discoideum
Source: PLoS Genet. 2011 Apr 28;7(4):e1002052. doi: 10.1371/journal.pgen.1002052 (PMC3084204; doi:10.1371/journal.pgen.1002052)
Supplement: Table S2 — Primers used to detect the presence of alternatively spliced hd mRNA transcripts during growth and development. Sequences of primers presented in the 5′-3′ direction that was used in RT-PCR reactions to detect the presence of alternatively spliced hd transcripts from wild-type AX3 purified total RNA. (DOC) [file pgen.1002052.s004.doc]

**Table S2. Primers used to detect alternatively spliced *hd* mRNA transcripts.**

| **Primer name** | **Primer Sequence (5’ – 3’)** |
| --- | --- |
| **HtE2-E3_F** | **ATAACATCCATTTGTAGATATCATCCAAG** |
| **HtE2-E3_R** | **GTTTTGGCCAACTATATTCATAAGGTG** |
| **HtE1-E3_F** | **GCTGAAGAAAGTTTAAATAGAAC** |
| **HtE1-E3_R** | GTTTTGGCCAACTATATTCATAAGGTG |
| **HtE3-E4_F** | **ATTATCATCATTGGATAGACAGAAATTCTC** |
| **HtE3-E4_R** | **GTTTGAATTTTTGATGCAAATTCAGATTCC** |
